# Adenovirus-Associated Epidemic Keratoconjunctivitis Outbreaks — Four States, 2008–2010

**Published:** 2013-08-16

**Authors:** Diane King, Barbara Johnson, Darlene Miller, Emily M. Landon, Aaron DeVries, Susan Fuller, Jane Harper, Ruth Lynfield, Karen Alelis, Patricia High, Joanne Wendolowski, Ellen Rudowski, Barbara Montana, Bruce Wolf, Mary Efstathiou, Timothy Doyle, Melissa Schaefer, Priti Patel, Dean Erdman, Xiaoyan Lu, Eileen Schneider

**Affiliations:** Florida Dept of Health; Bascom Palmer Eye Institute, Univ of Miami School of Medicine; Univ of Chicago Medical Center, Illinois; Minnesota Dept of Health; Bergen County Div of Health Svcs; Ocean County Health Dept; Hackensack Univ Medical Center; New Jersey Dept of Health and Senior Svcs; New Jersey Public Health and Environmental Laboratories; Career Epidemiology Field Officer Program, Office of Public Health Preparedness and Response; Div of Healthcare Quality Promotion, National Center for Emerging and Zoonotic Infectious Diseases; Div of Viral Diseases, National Center for Immunization and Respiratory Diseases, CDC

Epidemic keratoconjunctivitis (EKC) is a highly contagious, severe form of conjunctivitis (1). During 2008–2010, six unrelated EKC outbreaks associated with human adenovirus (HAdV) in four states were reported to CDC. In total, 411 EKC cases were identified in Florida, Illinois, Minnesota, and New Jersey. In each outbreak, health-care–associated transmission appeared to occur via ophthalmologic examination; however, community transmission was also documented. These outbreaks resulted in significant morbidity and cost resulting from the number of persons affected, duration of the outbreaks, and the temporary closure of a neonatal intensive-care unit (NICU) and several clinics. Clusters of EKC infections should be reported to the appropriate state or local health department. In settings where ophthalmologic care is provided, routine adherence to basic infection control measures and early implementation of enhanced outbreak control measures are essential to prevent HAdV transmission.

Worldwide, EKC is one of the most common eye infections, occurring in various health-care settings and in the community. Typically, EKC outbreaks last weeks to months and are characterized by a combination of health-care–associated and community transmission. Outbreak investigation, if done, often occurs late in the outbreak. HAdVs, especially serotypes 8 (HAdV-8), 19 (HAdV-19), and 37 (HAdV-37), are common etiologic agents of EKC. Risk factors identified in past outbreaks of EKC include common ophthalmologic procedures such as tonometry, slit lamp examinations, and contact lens placement, as well as contact with infected clinicians (1–3). Symptoms usually appear within 14 days after exposure and commonly include a gritty feeling in the eyes, watery discharge, photophobia, and redness. Corneal involvement, including keratitis and subepithelial infiltrates, often develops in patients within days and can persist for months, affecting visual acuity. Clinical illness typically lasts 7–21 days and is usually self-limited. Transmission is predominately through contact with infected eye secretions via contaminated surfaces, instruments, eye drops, or hands. A person can be infectious a few days before developing symptoms to approximately 14 days after symptom onset. Diagnosis is primarily based on clinical findings. Laboratory tests to detect HAdV in conjunctival specimens, such as viral culture or polymerase chain reaction (PCR) assays, are not routinely used in clinics. Currently, no vaccines or antiviral drugs are available, and treatment is supportive.

## Florida

In March 2009, the Florida Department of Health initiated an investigation after being informed of an EKC outbreak at an outpatient ophthalmology practice that consisted of two separate clinics that predominately served elderly patients (4). During November 2008–March 2009, 37 persons were clinically diagnosed with EKC, including the sole staff physician, who continued to work while symptomatic. Among those patients, 23 (62%) visited the ophthalmology practice within 17 days before onset of their symptoms. Eight (22%) patients developed keratitis requiring long-term topical steroid treatment. Conjunctival specimens collected for viral culture from four patients were all positive for HAdV-8. In March 2009, the practice was closed for 1 day for intensive cleaning. Additional interventions included discarding all reusable eye drop vials, reprocessing of tonometers, dedicating an examination room to conjunctivitis patients, cohorting patients in the waiting area, and disinfecting examination room surfaces after each patient visit (Table).

## Illinois

In March 2009, two premature infants in a NICU were found through laboratory testing to have EKC caused by HAdV-19. Case finding identified an additional 10 NICU infants with EKC. All 12 patients had been examined by the ophthalmologic team for retinopathy of prematurity (ROP) within the previous 34 days. Reusable scleral depressors and ocular specula were used for ROP examinations and soaked in isopropyl alcohol between use. Further investigation revealed six additional persons with EKC among NICU staff members (n = 2), patient family members (n = 2), and the ophthalmologic team (n = 2), including an ophthalmology resident who continued to work while symptomatic. During the investigation, an ophthalmologic equipment cart used to transport clean and used equipment was disinfected and stored in a closet unused. Nine days after disinfection, HAdV-19 was detected by PCR in eight of nine specimens collected from the cart, three of which were also HAdV-positive by viral culture. The NICU was closed to new admissions for 23 days. Contact and droplet precautions (5) were instituted in the unit, restriction of sick staff members and visitors was reinforced, medical and ophthalmologic equipment was cleaned and disinfected, surveillance was instituted, and the ROP examination protocol was updated to mandate the use of disposable ROP equipment to protect against future outbreaks (Table).

## Minnesota

In August 2008, the Minnesota Department of Health was contacted by local public health officials about a cluster of EKC patients in a rural setting. An investigation was initiated identifying 70 cases, including eight health-care staff members with EKC from three ophthalmology and optometry outpatient clinics. Symptom onset for these 70 cases occurred during June 28–September 25. Ten cases were laboratory confirmed for HAdV by viral culture or PCR, and three were typed and identified as HAdV-8. Among the infected patients, 33 had visited one of the three clinics within a median of 9 days (range: 3–21 days) before symptom onset. Many of the 70 patients with EKC developed significant morbidity (53% keratitis or corneal erosions, 41% membranous conjunctivitis, 40% decreased visual acuity) requiring additional and prolonged care. In August, all three affected clinics began implementing recommended infection control activities. Those included enhanced surveillance for additional cases, improved equipment reprocessing and environmental cleaning and disinfection, and cohorting of suspected conjunctivitis patients (Table).

## New Jersey

During December 2009–July 2010, three separate EKC outbreaks involving approximately 300 persons were documented. For all three outbreaks, cases in patients who visited an ophthalmology clinic within 30 days before onset or who were linked to a clinic case were considered facility-associated. The largest outbreak was reported from an ophthalmologic practice (one main clinic and four satellite clinics) where 245 persons with EKC were identified; 55% of those cases were facility-associated. HAdV-8 was detected by PCR in conjunctival swabs from three of four persons tested. The second and third outbreaks were reported in smaller ophthalmologic practices. In the second outbreak, 17 persons with EKC were identified, including one staff optometrist; eight cases were facility associated. HAdV-3 was detected by PCR in the three conjunctival swabs collected. In the third outbreak, 24 persons with EKC were identified; 13 were facility associated, and HAdV-8 was detected by PCR in conjunctival swabs from the six persons tested. Environmental samples collected in the first and second outbreaks were negative. In the third outbreak, HAdV was detected by PCR in three (slit lamp chin rest, slit lamp grab bar, and tonometer tip disinfection container) of nine environmental samples collected 4 days after infection control measures were implemented with the aim of improving hand hygiene and disinfection of equipment and surfaces. Viral culture, to confirm virus viability, was not performed. Recommended interventions for all of the clinics involved in these outbreaks included 1) improved staff hand hygiene, 2) environmental surface disinfection performed after every patient visit, 3) use of smaller eye drop vials to limit administration to multiple patients, 4) a separate examination room for suspected EKC patients, and 5) triaging of EKC patients in the waiting area (Table).

What is already known on this topic?

Human adenovirus (HAdV)–associated epidemic keratoconjunctivitis (EKC) is a highly contagious, severe form of conjunctivitis. Outbreaks of HAdV-associated EKC can result in significant morbidity through simultaneous health-care–associated and community transmission. HAdVs are viable for long periods in the environment and can be difficult to control.

What is added by this report?

During 2008–2010, six unrelated, HAdV-associated EKC outbreaks were reported to CDC. In total, 411 EKC cases were identified in four states. In each outbreak, health-care–associated transmission appeared to occur via ophthalmologic examination; however, community transmission was also documented. These outbreaks resulted in significant morbidity and cost because of the number of persons affected, duration of the outbreaks, and the temporary closure of health facilities.

What are the implications for public health practices?

In settings where ophthalmologic care is provided, increased awareness of EKC, routine adherence to basic infection control measures, and early implementation of enhanced outbreak control measures are essential to preventing HAdV transmission. Clusters of EKC infections should be reported to the appropriate state or local health department.

### Editorial Note

EKC outbreaks are common worldwide. In the United States, the prevalence and incidence of EKC is unknown. Although HAdV-associated EKC is not a reportable condition, most states have general reporting requirements concerning potential outbreaks. Suspected EKC outbreaks should be reported promptly to local and state public health authorities. Delays in identification and reporting of an EKC outbreak or institution of infection control measures can impede timely investigation and prolong transmission (6).

Control of EKC outbreaks is made more difficult because of prolonged shedding of HAdV, which can last several days to weeks after symptom resolution, and because of HAdV resistance to desiccation. HAdV is resistant to many common disinfectants and can remain viable for long periods in the environment (7–10). HAdV has been detected in ophthalmic solutions and on instruments and other environmental surfaces for >1 week after exposure. In the Illinois outbreak, HAdV was detected and cultured from ophthalmologic equipment >1 week after disinfection. In one of the New Jersey outbreaks, HAdV was detected on high-touch surfaces 4 days after clinic disinfection.

Isopropyl alcohol should not be relied on for disinfection of ophthalmologic instruments that contact mucous membranes or normally sterile body sites; use of 70% isopropyl alcohol has been associated with previous outbreaks of HAdV (3,10). Instead, equipment manufacturer’s instructions should be followed for disinfection or sterilization of all instruments. Facilities should use single-use disposable instruments whenever possible, especially in settings where adherence to recommended disinfection or sterilization practices cannot be ensured.

Many published health-care–associated EKC outbreaks have had simultaneous community transmission (1–3); however, prevention and control efforts have focused on health-care settings. Among the six outbreaks described in this report, five occurred in outpatient settings, health-care providers were likely sources of transmission in four, and infection control breaches were noted in all. Clinicians in all health-care settings are expected to follow basic infection control practices, including standard precautions (5). These expectations for outpatient settings are emphasized in a recent summary of CDC guidance.*

Outpatient clinics, hospitals, and other facilities that provide ophthalmologic care should have protocols in place to prevent transmission of EKC. Infection control measures used by the clinics before and after the outbreak varied. Measures that should be followed routinely include 1) strict adherence to hand hygiene among staff members, 2) use of disposable gloves for any potential contact with eye secretions, 3) disinfection of ophthalmic instruments after each use (or use of disposable equipment), 4) cohorting of suspected conjunctivitis patients (separate waiting room, sign-in area, and examination room), and 5) furloughing of staff members who have signs and symptoms consistent with EKC. Dedicating eye drop vials to single patients and increasing the frequency of environmental surface disinfection are strategies that should be used in outbreak situations and should be considered as routine practice to help prevent outbreaks.

## Figures and Tables

**TABLE:** Characteristics of six human adenovirus (HAdV)–associated epidemic keratoconjunctivitis (EKC) outbreaks — Florida, Illinois, Minnesota, and New Jersey, 2008–2010

Characteristic	Florida	Illinois	Minnesota	New Jersey
Year	2009	2009	2008	2009–2010
Health-care setting	Outpatient clinics (n = 2)	Neonatal intensive-care unit (n = 1)	Outpatient clinics (n = 3)	Outpatient clinics (n = 7)
No. of outbreaks	1	1	1	3
Total EKC cases	37	18	70	286
HAdV serotype identified	HAdV-8	HAdV-19	HAdV-8	HAdV-8, HAdV-3*
No. of facility-associated cases†	23	16	33	156
No. of health-care workers with EKC§	1	4	8	1
Infection control breach at facility¶	Yes	Yes	Yes	Yes
HAdV detected on medical equipment	Not tested	Yes	Not tested	Yes (in 1 of 3 outbreaks)
Control measures instituted**	Yes	Yes	Yes	Yes

* HAdV-3 was identified in one of the outbreaks, and HAdV-8 was identified in two of the outbreaks.

† Might represent a minimum case number.

§ Signs and symptoms consistent with EKC.

¶ Infection control breaches included poor hand hygiene and lack of cleaning and disinfection of shared medical equipment between patients.

** Control measures included extensive cleaning of the clinic and medical equipment, discarding reusable eye drop vials, replacing reusable eye drop vials with single-use eye drop vials, cohorting suspected conjuntivitis patients, improving hand hygiene techniques, instituting surveillance for EKC, and furloughing staff with suspected EKC.

## Footnotes

* Guidance available at http://www.cdc.gov/hai/settings/outpatient/outpatient-care-guidelines.html.
